# Overdiagnosis of ductal carcinoma in situ by grade and definition in population-based screening: A modeling study

**DOI:** 10.1016/j.breast.2025.104594

**Published:** 2025-10-10

**Authors:** Keris Poelhekken, Marcel J.W. Greuter, Bert van der Vegt, Monique D. Dorrius, Geertruida H. de Bock

**Affiliations:** a University of Groningen, University Medical Center Groningen, Department of Epidemiology, Groningen, P.O. Box 30 001, FA40, Groningen, 9700 RB, the Netherlands; b University of Groningen, University Medical Center Groningen, Department of Radiology, Groningen, PO Box 30.001, EB44, Groningen, 9700 RB, the Netherlands; c University of Groningen, University Medical Center Groningen, Department of Pathology and Medical Biology, Groningen, PO Box 30.001, Groningen, 9700 RB, the Netherlands

**Keywords:** Breast neoplasms, Breast carcinoma in situ, Early detection of cancer, Computational modeling, Disease progression

## Abstract

**Aim:**

To estimate ductal carcinoma in situ (DCIS) overdiagnosis overall and by grade in population-based screening and to determine the variation in overdiagnosis estimates by definition.

**Methods:**

Using a fully validated micro-simulation Markov model for DCIS (SimDCIS), the number, rate, and proportion of DCIS overdiagnoses were estimated overall and by grade. Overdiagnoses comprised excess DCIS cases in the screened versus the unscreened population; overdiagnosis rate equaled the number of DCIS overdiagnoses per 100,000 screened women; and DCIS overdiagnosis proportion equaled overdiagnosed DCIS divided by total diagnosed DCIS in the screened population. Base estimates for overdiagnosed DCIS were from a population perspective (ages 50–100 years) and included screen-detected, clinically detected, or progressed DCIS (i.e., invasive breast cancer with DCIS precursor). Overdiagnosis was also estimated for alternative definitions and perspectives. Univariate and probabilistic sensitivity analyses were performed to estimate uncertainty.

**Results:**

Base definitions yielded an overdiagnosis rate of 38.1 (range, 25.7–58.7) per 100,000 screened women and a proportion of 20 % (range 13 %–30 %). Stratification by grade showed 24 %, 20 %, and 18 % proportion overdiagnosis for grades 1, 2, and 3, respectively. Varying the definition led to overdiagnosis estimates from 18 % to 94 %; these overdiagnosis estimates increased by 36 %–49 % when excluding invasive breast cancer and by 54 %–71 % when including only screen-detected DCIS. Individual perspective estimates were 12 % higher than population perspective estimates.

**Conclusion:**

In biennial screening, approximately 1 in 5 DCIS is overdiagnosed, but with minimal variation between grades. A consensus definition and perspective for overdiagnosis would reduce the observed variation in DCIS overdiagnosis estimates.

## Highlights

• In biennial screening of women aged 50–75, 1 in 5 DCIS cases is overdiagnosed.
• Overdiagnosis rates are 24 %, 20 %, and 18 % for DCIS grade 1, 2, and 3, respectively.
• Overdiagnosis estimates vary widely (18 %–94 %) based on definition.
• Excluding DCIS progression to IBC overestimates overdiagnosis by 36 %–49 %.
• Excluding IBC and clinical detection overestimates overdiagnosis by 54 %–71 %.

## 1 Introduction

Ductal carcinoma in situ (DCIS) accounts for approximately 25 % of all breast cancers detected in countries with population-based screening [1]. Diagnosis has increased markedly since the implementation of screening, due in part, to the increased sensitivity of mammography [2]; in turn, this has led to concerns of overdiagnosis and overtreatment [1,[3], [4], [5]]. Overdiagnosis is the detection of breast cancer that would not have been diagnosed during a woman's life without screening [5], whereas overtreatment occurs when a patient receives treatment that does not reduce breast cancer mortality [4]. Given that DCIS is always treated when diagnosed, all overdiagnosis results in overtreatment. Other relevant consequences of overdiagnosis include the financial and psychological burdens associated with unnecessary biopsy, fear to be the unlucky one, and stress in anticipation of hospital appointments [6,7].

Estimates of DCIS overdiagnosis range from 20 % to 91 % [4], and it is considered the largest contributor to breast cancer overdiagnosis [1,8]. However, these estimates are influenced by the definition of overdiagnosis [[9], [10], [11], [12]]. There is wide variation in literature on what is included as overdiagnosis [8,10,13,14], the perspective (e.g., individual or population-based) [11,15,16], and the reported measures [9,10,17] for DCIS (Table 1). Furthermore, immediate treatment of DCIS results in uncertainty around its natural history because progression and regression cannot be observed [4], leading to assumptions in research that further obscure true estimates of overdiagnosis [4,6,9,12,18]. Over the past 10 years more studies on the natural history and risk factors associated with DCIS have emerged [18], providing insight into the processes and risk factors associated with progression from DCIS to invasive breast cancer (IBC) and regression (e.g., younger age and DCIS grade) [7,10,15,19]. DCIS is commonly divided into three grades linked to its potential to progress to IBC [20]. Given increases in progression and regression are associated with high grade DCIS, it is important to consider DCIS grade in estimates of overdiagnosis [4,21].

**Table 1:** Variations in definition of overdiagnosis estimates for DCIS.

Included in definition of DCIS overdiagnosis	Reference
**Overdiagnosed DCIS**	
Screen-detected, clinical detected, progressed to IBC	[8]
Screen-detected, clinical detected	[10,13]
Screen-detected	[14]
**Perspective**	
Individual (start screening to end screening)	[11]
Population (start screening to death)	[11,16]
Lifetime (birth to death)	[15]
**Reported measure**	
Number overdiagnosed	[9]
Overdiagnosis rate	[9,16,24]
Overdiagnosed proportion	[10,14,16,17]

Overview of variations in definitions identified in literature. Abbreviations: DCIS, ductal carcinoma in situ; IBC, invasive breast cancer (specifically, DCIS progressed to IBC).

The first randomized controlled trials have been set up to observe the natural course of low grade DCIS (i.e., grades 1 and 2), but these will not only take time to provide results due to strict inclusion criteria [4,7] but will also not provide an overall estimate of overdiagnosis [4]. Simulation models can overcome these limitations to estimate overdiagnosis accurately, provided that the models are fully validated, uncertainty is adequately assessed, and assumptions are well reported [17,21]. A recent review reported overdiagnosis of up to 65 % in previous modeling studies [18]. Five models from the Cancer Intervention and Surveillance Modeling Network (CISNET) have estimated the proportion of overdiagnosed DCIS to be between 34 % and 72 % [8,16]. Another simulation study estimated overdiagnosis for DCIS grades 1, 2, and 3 to be 60 %, 56 %, and 45 %, respectively [10]. However, that study did not provide an overall estimate of DCIS overdiagnosis and only used data up to 2010. Between 2005 and 2010, a transition from screen-film to digital mammography took place that may have affected DCIS detection [2,22]. A new micro-simulation Markov model for the natural history of DCIS (SimDCIS) was recently developed and fully validated. It has shown the ability to provide accurate, grade-dependent estimates of population-based screening [23].

The aim of this study was to estimate DCIS overdiagnosis overall and stratified by grade, and to address the gap in comparative methodology by exploring the variation in overdiagnosis estimates by definition, using the SimDCIS model.

## 2 Methods

### 2.1 SimDCIS in the Dutch screening setting

The SimDCIS model was applied to Dutch setting, where breast cancer screening has been offered since 1989–1998 [25]. All women aged 50–75 years without a history of breast cancer are invited for biennial mammographic examination, with compliance of 76 % reported in 2019 [25]. SimDCIS has been validated for use in the Dutch screening setting [23].

Input parameters include four transition probabilities (i.e., healthy to death, healthy to DCIS, DCIS to healthy, DCIS to IBC), four screening parameters (i.e., sensitivity, age, frequency, participation), and a clinical detection parameter. A detailed explanation of the SimDCIS model was published previously [23]. All input parameters for the Dutch screening setting were independently derived from literature, resulting in no parameter fitting to observed data [23].

In each simulation, a virtual cohort of 100,000 women was created and averaged over 10 cohorts to mimic true women in the Netherlands. Each woman was followed from birth to either death, screen-detected DCIS, clinically detected DCIS, or IBC. The probability of death was age-dependent and based on Dutch data from the Central Bureau of Statistics [26]. DCIS without progression does not change the probability of death, which was therefore set as equal to that for healthy women [15,21]. Each woman was assigned an age- and grade-dependent probability of developing DCIS in her lifetime based on age- and grade-dependent data from the Netherlands Cancer Registry for 2015–2022 (excluding 2020, due to data fluctuations that resulted from the COVID-19 pandemic) [27].

DCIS has the probability of regression, detection, and age- and grade-dependent progression to IBC. Regression of DCIS was possible to the healthy state independent of age and grade and was set to 5 % [21,28]. Detection could be through either mammography screening or clinical detection, with sensitivity set to 86 % [29] and 5 % [10], respectively. Progression to IBC was based on age-dependent data from the US National Cancer Institute's Surveillance, Epidemiology, and End Results program of 1992–2014 [30]. Direct progression from healthy state to IBC (without DCIS as a precursor) was considered possible but was outside the scope of this model because direct progression does not affect DCIS overdiagnosis (i.e., IBC without a DCIS precursor is not used to calculate DCIS overdiagnosis).

### 2.2 Base definitions

The main model outcomes were the number, rate, and proportion of overdiagnosed DCIS (Appendix A.1.) estimated overall and stratified by DCIS grade. To determine the number of overdiagnosed DCIS, the total number of diagnosed DCIS in the non-screened cohort (DCIS clinically detected or progressed to IBC) was subtracted from the number of DCIS diagnosed in a screened cohort (screen-detected, clinically detected, or progressed to IBC). Clinical detection and progression to IBC were both included in the calculation because non-symptomatic and non-progressive DCIS can both lead to overdiagnosis [8]. Consequently, it was assumed that all DCIS progressing to IBC will be detected; in addition to screen and clinical detection, each progression of DCIS to IBC was therefore counted as diagnosed DCIS. Overdiagnosis rate was calculated as the number of overdiagnosed DCIS per 100,000 screened women. The proportion of overdiagnosed DCIS was calculated as the number of overdiagnosed DCIS divided by the number of diagnosed DCIS in the screened population.

For all simulations, screening consisted of biennial screening from age 50–75. The non-screened and screened cohort comprised identical women to ensure that output variation was only due to screening implementation. Women were followed from screening start (age 50 years) to the end of follow-up (death or breast cancer diagnosis), referred to as the population perspective.

### 2.3 Alternative definitions

**Fig. 1:**
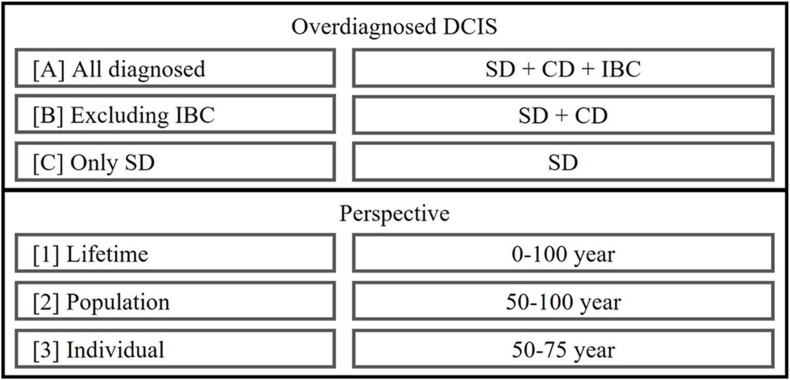
Definition variations for the proportion of overdiagnosed DCIS. Definition of proportion overdiagnosed DCIS was varied by overdiagnosis (A, B, C) and perspective (1, 2, 3). The base definition (A2) combined all diagnosed (screen-detected, clinically detected, and progressed to IBC) from a population perspective (diagnosed DCIS in women 50–100 years old). All proportions were calculated as the number of overdiagnosed DCIS: [diagnosed in screening – diagnosed without screening]/[total number of DCIS diagnosed in screening]. Abbreviations: DCIS, ductal carcinoma in situ; CD, clinically detected DCIS; IBC, invasive breast cancer (specifically, DCIS progressed to IBC); SD, screen-detected DCIS.

Analysis was repeated using alternative definitions for both overdiagnosis (definitions A, B, C) and the perspective (definitions 1, 2, 3) and compared to base definition (definition A2) (Fig. 1). The alternative definitions were based on the variation in the definitions of overdiagnosis in previous literature [8,11]. IBC was excluded in definition B and clinically detected DCIS and IBC were both excluded in definition C (Appendix A.2.). Concerning the perspective, overdiagnosis estimates were made from the individual perspective (definition 1; including women from the start to the end of screening), from the population perspective (definition 2; the base definition), and from lifetime perspective (definition 3; including women from birth to end of follow-up) [10,11,15,16]. In addition, overdiagnosed DCIS was estimated by including only screen- and clinically detected DCIS (Appendix A.3.).

### 2.4 Sensitivity analyses

Univariate sensitivity analyses were performed to evaluate robustness of the overdiagnosis estimate. For each input parameter, lower and upper 95 % confidence intervals (95 %CI) were applied (Appendix A.4.) and the change in proportion of overdiagnosed DCIS from the base definition was recorded. Results are summarized in a tornado plot. A probabilistic sensitivity analysis was performed to evaluate overall uncertainty of the overdiagnosis estimates in 100 Monte-Carlo simulations with random selection within the 95 %CI of all input parameters. For each Monte-Carlo simulation, a non-screened cohort with identical input was simulated to calculate the proportion of overdiagnosed DCIS.

## 3 Results

### 3.1 Overdiagnosis estimation

Using the base definition, the overdiagnosis rate was estimated at 38.1 per 100,000 screened women (Table 2). The proportion of overdiagnosed DCIS (definition A2) was estimated at 20 %, and when stratified by grade, was estimated at 24 %, 20 %, and 18 % for grades 1, 2, and 3, respectively.

**Table 2:** Overdiagnosis of DCIS.

Grade	Number of DCIS overdiagnosed	DCIS overdiagnosis rate /100,000 screened	Proportion DCIS overdiagnosed
Total	340 (197–456)	38.1 (22.7–51.1)	20 % (11 %–28 %)
1	78 (48–111)	8.8 (5.5–12.3)	24 % (14 %–34 %)
2	131 (74–172)	14.7 (8.4–19.4)	20 % (11 %–27 %)
3	131 (73–179)	14.7 (8.3–19.6)	18 % (10 %–25 %)

DCIS number, rate, and proportion overdiagnosis totals and stratification by DCIS grade from a population perspective. DCIS overdiagnoses are calculated as: [sum of all screen-detected, clinically detected, and IBC progression among screening cases] – [sum in non-screening cases]. Proportions were calculated as: [diagnosed with screening – diagnosed without screening]/[total number of DCIS diagnosed with screening]. Abbreviations: DCIS, ductal carcinoma in situ; IBC, invasive breast cancer (specifically, DCIS progressed to IBC).

### 3.2 Definitions

**Fig. 2:**
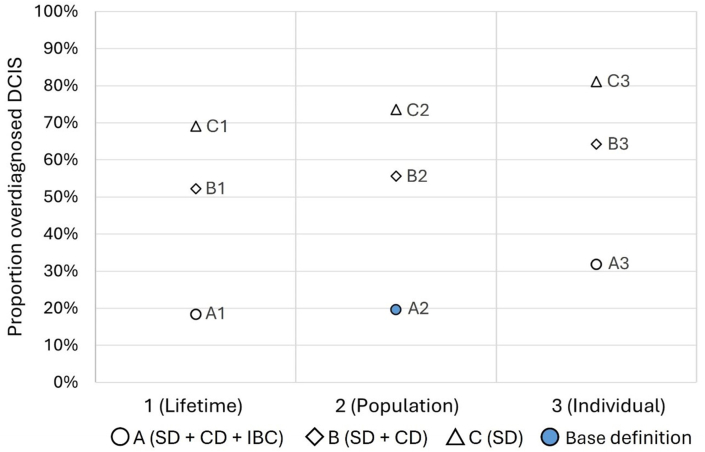
Estimated proportion of DCIS overdiagnosed by variation in definition. Variation in the estimated proportion of overdiagnosed DCIS for definitions A, B, C, from perspectives 1, 2, 3. The base definition A2 is marked (blue). All proportions were calculated as number of overdiagnosed DCIS ([diagnosed with screening – diagnosed without screening]/[total number of DCIS diagnosed with screening]) including IBC. Shape indicates type of detection included as overdiagnosed. 95 % Confidence intervals were reported in Appendix A.3.1. Abbreviations: DCIS, ductal carcinoma in situ; CD, clinically detected DCIS; IBC, invasive breast cancer (specifically, DCIS progressed to IBC); SD, screen-detected DCIS. (For interpretation of the references to colour in this figure legend, the reader is referred to the Web version of this article.)

Variation in definition resulted in a proportion of 18 %–94 % overdiagnosed DCIS (Fig. 2). Compared to the base definition A2, definition B2 and C2 were associated with 36 % and 54 % increases in the proportion of overdiagnosed DCIS, respecitvely. Compared to estimates from a population perspective, those from individual perspective (i.e., A3, B3, C3) were up to 12 % higher and those from a lifetime perspective (i.e., A1, B1, C1) were 1 %–5 % lower. Overdiagnosis estimates that excluded DCIS progression to IBC increased by 5 %–20 % compared with estimates that included progression to IBC (Appendix A3.3.).

### 3.3 Sensitivity analyses

**Fig. 3:**
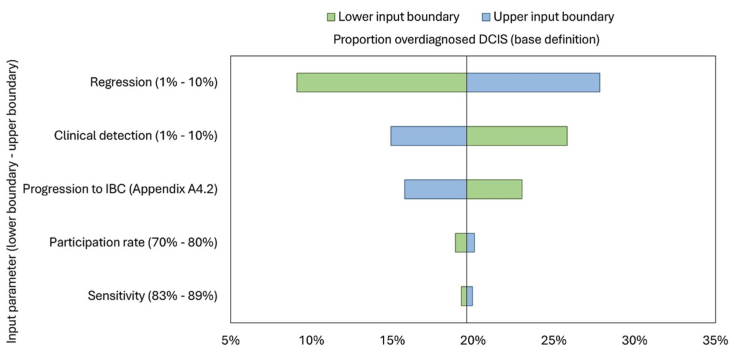
Univariate sensitivity analyses for proportion of DCIS overdiagnosed. Robustness of the proportion of overdiagnosed DCIS, calculated in the following base definition: [diagnosed with screening – diagnosed without screening]/[total number of DCIS diagnosed with screening]. Abbreviations: DCIS, ductal carcinoma in situ; IBC, invasive breast cancer (specifically, DCIS progressed to IBC).

**Fig. 4:**
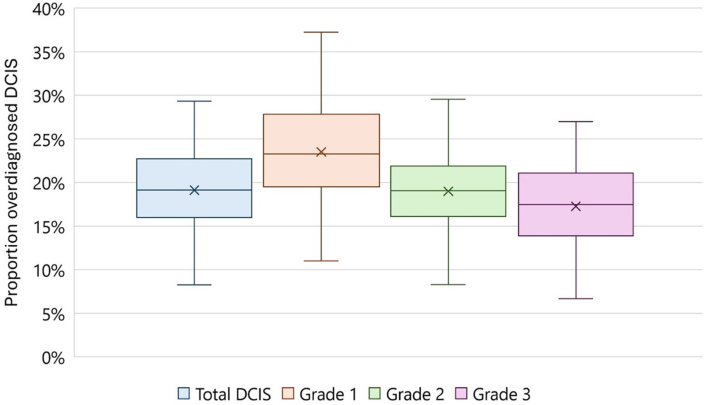
Probabilistic sensitivity analysis for proportion of DCIS overdiagnosed. Overall uncertainty of the proportion of overdiagnosed DCIS derived from 100 Monte Carlo simulations. Proportions were calculated in the following base definition: [diagnosed with screening – diagnosed without screening]/[total number of DCIS diagnosed with screening]. Abbreviations: DCIS, ductal carcinoma in situ; IBC, invasive breast cancer (specifically, DCIS progressed to IBC).

Univariate sensitivity analyses for the base definition of the proportion of overdiagnosed DCIS ranged from 9 % to 28 % (Fig. 3). Changes in regression and clinical detection resulted in the largest variations in the overdiagnosed proportion (9 %–28 % and 15 %–26 %, respectively). The probabilistic sensitivity analysis showed a maximum variation of 14 % in the proportion of overdiagnosed DCIS (Fig. 4).

## 4 Discussion

DCIS overdiagnosis was estimated at 20 % using SimDCIS in the Dutch screening setting; when stratified by grade, DCIS overdiagnosis was estimated at 24 %, 20 %, and 18 % for grades 1, 2, and 3, respectively. Varying the definition of overdiagnosis led to the proportion overdiagnosed DCIS varying from 18 % to 94 %. The proportion of overdiagnosed DCIS increased by up to 36 % when excluding DCIS progression to IBC, by up to 54 % when including screen-detected tumors only, and by up to 12 % when analyzing data from an individual perspective. Sensitivity analyses showed change in the probability of regression and clinical detection had the largest impact on model output, resulting in maximum variation of 14 %.

DCIS overdiagnosis estimates were given within the previously reported range of 20 %–91 % [4]. A recent review of modeling studies reported an estimated overdiagnosis of up to 65 % [18], while five models from CISNET estimated the proportion of overdiagnosed DCIS at 34 %–72 % [8,16]. Using our base definition, 1 in 5 cases of DCIS is overdiagnosed by biennial screening. One study estimated DCIS overdiagnosis from a population perspective of 60 %, 56 %, and 45 % for grades 1, 2, and 3, respectively [10]; our corresponding estimates of 24 %, 20 %, and 18 % are much lower. Although both estimates were derived from a population perspective, the overdiagnosis estimates produced by van Luijt et al. did not include progression to IBC. Using the same definition (excluding IBC) in our study resulted in a similar overdiagnosis estimate of 56 % [10]. Both studies found increased overdiagnosis for grade 1 compared to 3, consistent with the biological characteristics of high grade DCIS increasing progression to IBC [3,4]. To determine the difference in overdiagnosis between DCIS grades, improved knowledge is needed of the differences in regression and progression between grades. At 20 %, the overdiagnosis estimate for our base definition was toward the lower end of previous estimates.

Variations in the definition of overdiagnosis can explain observed differences in previously reported estimates. The US Preventive Services Task Force (USPSTF) recommends including both clinical detection and DCIS that has progressed to IBC in the definition, because both non-progressive DCIS and not presenting clinically are causes of overdiagnosis [8]. In the literature, however, estimates are reported without considering progression to IBC [10,13] or only include screen-detected DCIS [14]. In our study, excluding DCIS that progressed to IBC from the definition of overdiagnosis resulted in an estimated 56 % overdiagnosis, whereas completely excluding progression to IBC resulted in an estimated 69 % overdiagnosis. Using only screen-detected DCIS led to 74 % overdiagnosis, which is consistent with the results of previous studies that included only screen-detected tumors [14]. This latter definition assumes that screen-detected DCIS will not cause symptoms, will not progress to IBC, and will not lead to breast cancer-related death. Often, DCIS is seen as not contributing to morbidity or mortality, but screen-detected DCIS could still be detected clinically or progress without screening, resulting in an overestimation of DCIS overdiagnosis. Therefore, progression to IBC and clinical detection should both be included in estimates of DCIS overdiagnosis.

The perspective taken when estimating overdiagnosis can also explain part of the observed variation. Although most estimates are made from a population perspective, an independent review panel from the UK recommended using both individual and population perspectives [11], but in literature lifetime perspective was also used [15,16]. We found that estimates from a lifetime perspective were very similar to those from a population perspective, with a change of only −1 % to −5 % in overdiagnosis estimates. This small change results from two factors. First, the number of overdiagnosed DCIS was the same from population and lifetime perspectives because the model is identical for the cohort with and without screening, except for the introduction of screening, which means the DCIS diagnosis was the same before screening started. Second, the total number diagnosed only slightly increased from lifetime perspective because DCIS was hardly diagnosed without screening before age 50 years. The individual perspective increased estimates by 8 %–12 %, giving a more personalized view of the probability that DCIS during the screening window is overdiagnosed. However, follow-up might be too short and lead to overestimates of overdiagnosis, especially for tumors diagnosed at the end of screening. The population perspective has adequate follow-up for all tumors and is recommended for overdiagnosis estimates.

The large variation in DCIS overdiagnosis estimates in previous studies did not result solely from differences in the definition of overdiagnosis. Estimates from five CISNET models showed a range from 34 % to 72 % when using the same definition, partly due to variations in assumptions that were not always clearly reported [8,16,22]. Different assumptions regarding the natural history will lead to different overdiagnosis estimates [31]. If all IBC have a DCIS precursor, approximately 61 %–91 % of DCIS is estimated to progress, but if IBC can occur without a DCIS precursor, estimates are markedly lower at around 20 %–25 % [16]. Recent evidence supports lower progression estimates because the direct formation of IBC without DCIS as a precursor is believed possible [7,21,32,33]. Using SimDCIS, approximately 20 % of DCIS were estimated to progress to IBC [23]. Univariate sensitivity analyses showed that both regression of DCIS and progression to IBC affected overdiagnosis estimates. Moreover, model structure and input data have varied among models; for example, mammographic sensitivity estimates range from 40 % to 99 % [16,32] and DCIS onset can rely on data from before or after transition to digital mammography [2,24]. Screening parameters such as age and frequency are also expected to affect overdiagnosis estimates [9,10,[34], [35], [36], [37], [38]], and it is important to consider lead-time and to ensure enough follow-up [7,13]. For IBC, follow-up time should be at least 10 years [9], but for DCIS, the adequate follow-up duration has not been established. Definition, assumptions, natural history, model structure and input, screening program, and follow-up time are all important factors to consider when estimating DCIS overdiagnosis.

Several strengths and limitations of this study should be considered. First, SimDCIS was suitable to make reliable overdiagnosis estimates of DCIS. However, the model required real-world data and was limited by the availability and quality of existing data, necessitating assumptions about DCIS onset, regression, and progression to IBC. Transition probabilities were derived from observed data, which introduced uncertainty because the data on regression were limited and undetected DCIS could not be taken into account. In addition, misclassification of DCIS grade could affect the grade dependency of transition probabilities and result in larger or smaller differences in overdiagnosis between grades [39,40]. Second, the model was previously fully validated and tested for robustness in Dutch and UK screening settings and was shown to produce reliable results. Although the model was thoroughly validated, grade-specific DCIS overdiagnosis was not validated due to a lack of real-world data. Given that overdiagnosis estimates were made in the context of screening, these estimates are not applicable to areas without screening. Although overdiagnosis estimates may vary with screening setting, variations in overdiagnosis caused by definition are expected to be generalizable. Third, model input was age- and grade-dependent, independently derived from literature, and included all natural history parameters within expected ranges. However, the model does simplify the truth by excluding other individual risk factors, such as mammographic density, hormonal factors, and family history. Fourth, this is the first study to estimate overdiagnosis both overall and stratified by DCIS grade. In the absence of a consensus definition for overdiagnosis, we chose a base definition that assumed all IBC with a DCIS precursor will be diagnosed. This assumption may have caused a slight underestimation of overdiagnosis, because not all IBC might be detected. In countries with well-implemented screening and high participation, this effect is expected to be negligible given that most IBC will be detected.

## 5 Conclusion

This study has clarified the influence of variations in definition on overdiagnosis estimates and provides recommendations to help reach a consensus on the definition of overdiagnosis. Approximately one in five cases of DCIS is overdiagnosed in the Dutch screening setting, with minimal difference between grades. Variation in the definition of overdiagnosed DCIS markedly influenced overdiagnosis estimates and can explain the large range of overdiagnosis estimates reported in literature. Consensus should be pursued for a definition of overdiagnosis. Consistent with previous recommendations, estimates of DCIS overdiagnosis should account for progression to IBC and should be made from population or individual perspective. Overdiagnosis studies should report their methods and assumptions transparently because the natural history strongly influences estimates. Anticipated results from randomized controlled trials might provide insights to improve assumptions made about the natural history of DCIS. In the meantime, however, modeling studies could be used to clarify the effects of follow-up time and screening program on overdiagnosis estimates. Improved knowledge of overdiagnosis and its influencing factors will allow policy makers and women to make informed decisions about screening.

## CRediT authorship contribution statement

**Keris Poelhekken:** Writing – review & editing, Writing – original draft, Visualization, Validation, Software, Resources, Project administration, Methodology, Investigation, Formal analysis, Data curation, Conceptualization. **Marcel J.W. Greuter:** Writing – review & editing, Writing – original draft, Visualization, Validation, Supervision, Software, Resources, Project administration, Methodology, Investigation, Formal analysis, Data curation, Conceptualization. **Bert van der Vegt:** Writing – review & editing, Writing – original draft, Visualization, Resources, Investigation, Formal analysis. **Monique D. Dorrius:** Writing – review & editing, Writing – original draft, Visualization, Validation, Supervision, Resources, Methodology, Investigation, Formal analysis. **Geertruida H. de Bock:** Writing – review & editing, Writing – original draft, Visualization, Validation, Supervision, Software, Resources, Project administration, Methodology, Investigation, Formal analysis, Data curation, Conceptualization.

## Ethical approval

This study used publicly available anonymized data; thus, it was exempt from the need for ethical approval (IRB approval no. M24.342520, Medical Ethics Review Board Groningen, the Netherlands).

## Declaration of competing interest

The authors declare the following financial interests/personal relationships which may be considered as potential competing interests: Keris Poelhekken reports writing assistance was provided by Dr Robert Sykes. Bert van der Vegt reports a relationship with Visiopharm and Philips and MSD Merck and Daiichi-Sankyo AstraZenica that includes: consulting or advisory. Bert van der Vegt reports a relationship with Visiopharm and Philips and MSD Merck that includes: speaking and lecture fees. Bert van der Vegt reports a relationship with OWKIN and GE Healthcare that includes: funding grants. Bert van der Vegt reports a relationship with DEKRA that includes: funding grants. If there are other authors, they declare that they have no known competing financial interests or personal relationships that could have appeared to influence the work reported in this paper.

## Data Availability

SimDCIS model v02 was used and is publicly available in GitHub (https://github.com/kp-gith/SimDCIS) in programming language C++.
