# Supplementary material for: Overdiagnosis of ductal carcinoma in situ by grade and definition in population-based screening: A modeling study
Source: Breast. 2025 Oct 10;84:104594. doi: 10.1016/j.breast.2025.104594 (PMC12547802; doi:10.1016/j.breast.2025.104594)
Supplement: Multimedia component 1 [file mmc1.docx]

Appendix

*Supplementary material for ‘Overdiagnosis of ductal carcinoma in situ by grade and definition in population-based screening: a modelling study’.*

Content

[*A.1. Overdiagnosis estimation: calculation main outcomes 2*](#_Toc203054106)

[*A.1.1. Diagnosed DCIS 2*](#_Toc203054107)

[*A.1.2. Number of overdiagnosed DCIS 2*](#_Toc203054108)

[*A.1.3. Rate of overdiagnosed DCIS 2*](#_Toc203054109)

[*A.1.4. Proportion of overdiagnosed DCIS 2*](#_Toc203054110)

[*A.2. Definition variation overdiagnosed 3*](#_Toc203054111)

[*A.2.1. Definition A overdiagnosed: SD + CD + IBC 3*](#_Toc203054112)

[*A.2.2. Definition B overdiagnosed: SD + CD 3*](#_Toc203054113)

[*A.2.3. Definition C overdiagnosed: SD 3*](#_Toc203054114)

[*A.3. Overdiagnosed proportion 4*](#_Toc203054115)

[*A.3.1. Variation in estimated proportion with 95%CI: IBC included 4*](#_Toc203054116)

[*A.3.2. Definition overdiagnosed: IBC excluded 4*](#_Toc203054117)

[*A.3.3. Variation in estimated proportion: IBC excluded 5*](#_Toc203054118)

[*A.3.4. Variation in estimated proportion with 95%CI: IBC excluded 5*](#_Toc203054119)

[*A.4. Univariate sensitivity analyses input 6*](#_Toc203054120)

[*A.4.1. Onset DCIS 6*](#_Toc203054121)

[*A.4.2. DCIS progression to IBC 6*](#_Toc203054122)

## A.1. Overdiagnosis estimation: calculation main outcomes

### A.1.1. Diagnosed DCIS

$$Diagnosed =Screen detected+Clinically detected+DCIS progressed to IBC$$

***** in no screen scenario formula reduces to CD + IBC.

###

### A.1.2. Number of overdiagnosed DCIS

$$Overdiagnosed = Diagnosed in screen scenario-Diagnosed in no screen scenario = \left[ SD+CD+IBC \right]screen-\left[ CD+IBC \right]no screen$$

with SD = screen-detected, CD = clinically detected, and IBC = DCIS progressed to IBC.

###

### A.1.3. Rate of overdiagnosed DCIS

$$Overdiagnosis rate =\frac{Number of overdiagnosed DCIS}{Number of screened women}*100,000= \frac{\left[ SD+CD+IBC \right]screen-\left[ CD+IBC \right]no screen}{Number of mammograms}*100,000$$

with SD = screen-detected, CD = clinically detected, and IBC = DCIS progressed to IBC.

###

### A.1.4. Proportion of overdiagnosed DCIS

$$Overdiagnosed proportion =\frac{Number of overdiagnosed DCIS}{Diagnosed DCIS screen scenario}*100\%= \frac{\left[ SD+CD+IBC \right]screen-\left[ CD+IBC \right]no screen}{\left[ SD+CD+IBC \right]screen} *100\%$$

with SD = screen-detected, CD = clinically detected, and IBC = DCIS progressed to IBC.

## A.2. Definition variation overdiagnosed

### A.2.1. Definition A overdiagnosed: SD + CD + IBC


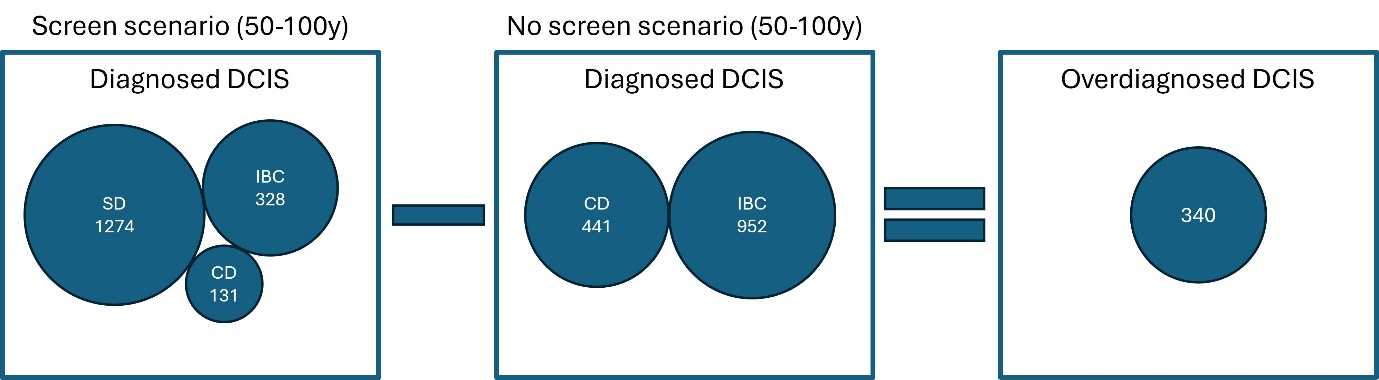


*Definition of overdiagnosed includes DCIS that is screen-detected (SD), clinically detected (CD), and progressed to IBC (IBC). Numbers indicate the number of diagnoses in that category, based on Dutch biennial screening setting versus no screening from a population perspective.*

### A.2.2. Definition B overdiagnosed: SD + CD


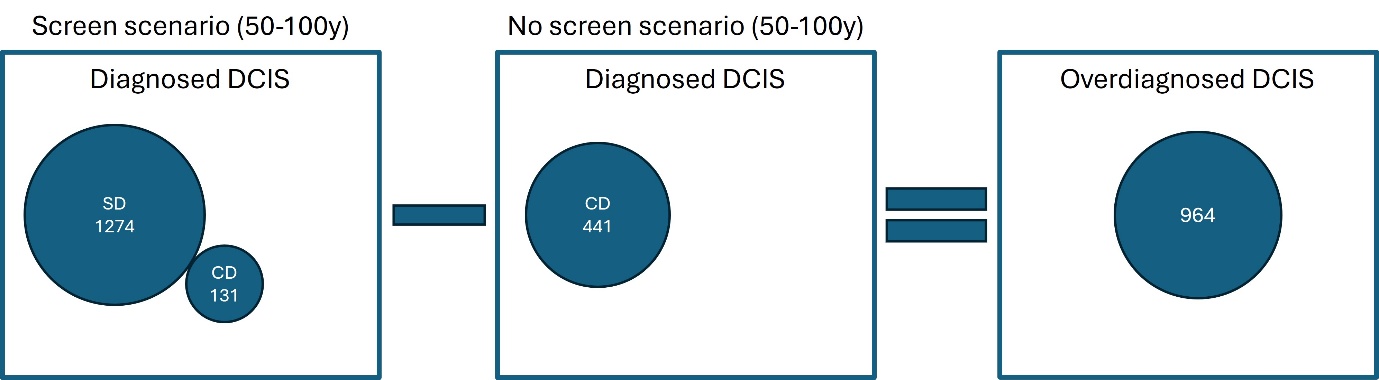


*Definition of overdiagnosed includes DCIS that is screen-detected (SD) and clinically detected (CD). Numbers indicate the number of diagnoses in that category, based on Dutch biennial screening setting versus no screening from a population perspective.*

### A.2.3. Definition C overdiagnosed: SD


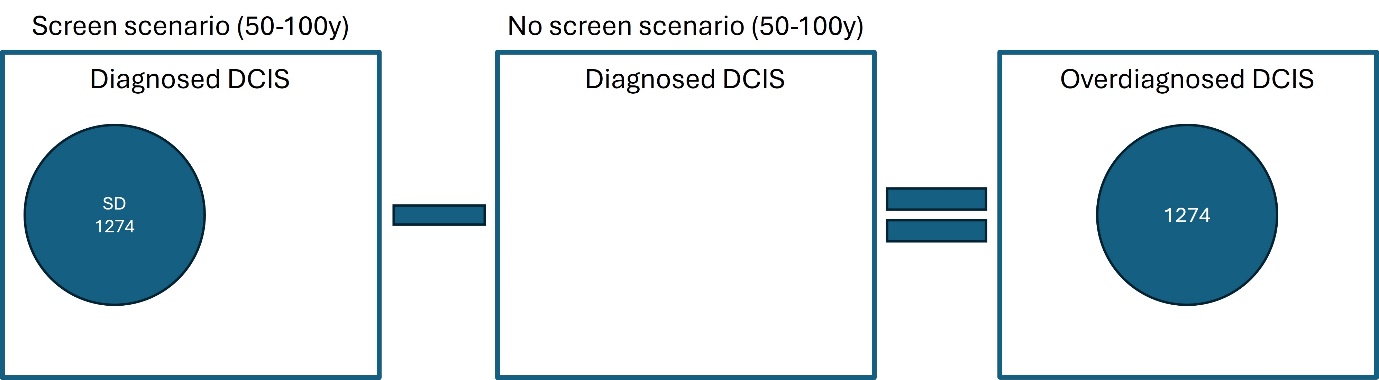


*Definition of overdiagnosed includes DCIS that is screen-detected (SD). Numbers indicate the number of diagnoses in that category, based on Dutch biennial screening setting versus no screening from a population perspective*.

## A.3. Overdiagnosed proportion

### A.3.1. Variation in estimated proportion with 95%CI: IBC included

| Proportion overdiagnosed DCIS (95%CI)–Data Figure 2 | | | |
| --- | --- | --- | --- |
| Definition: Overdiagnosed DCIS | Perspective | | |
|  | 1 (Lifetime) | 2 (Population) | 3 (Individual) |
| A (SD + CD + IBC) | 18% (10%–26%) | 20% (11%–28%) | 32% (23%–39%) |
| B (SD + CD) | 52% (42%–64%) | 56% (45%–67%) | 64% (54%–75%) |
| C (SD) | 69% (65%–72%) | 74% (70%–76%) | 81% (77%–83%) |

*Variation in the proportion overdiagnosed DCIS with 95% confidence intervals (addition to Figure 2). All proportions were calculated as number of overdiagnosed DCIS (diagnosed with screening–diagnosed without screening) divided by the total number of DCIS diagnosed with screening (including IBC). *DCIS = ductal carcinoma in situ, SD = screen-detected, CD = clinically detected, IBC = DCIS progressed to invasive breast cancer.*

###

### A.3.2. Definition overdiagnosed: IBC excluded


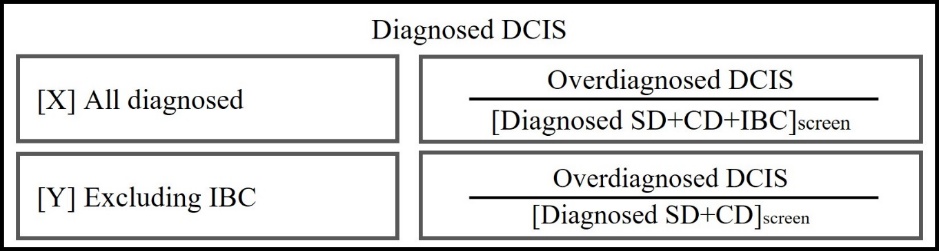


*Variation in definition of proportion overdiagnosed ductal carcinoma in situ (DCIS), excluding IBC. All proportions were calculated as number of overdiagnosed DCIS (diagnosed in scenario with screening–diagnosed in scenario without screening) divided by the total number of DCIS diagnosed in the screening scenario. *SD = Screen-detected DCIS; CD = clinically detected DCIS; IBC = DCIS progressed to invasive breast cancer.*

### A.3.3. Variation in estimated proportion: IBC excluded


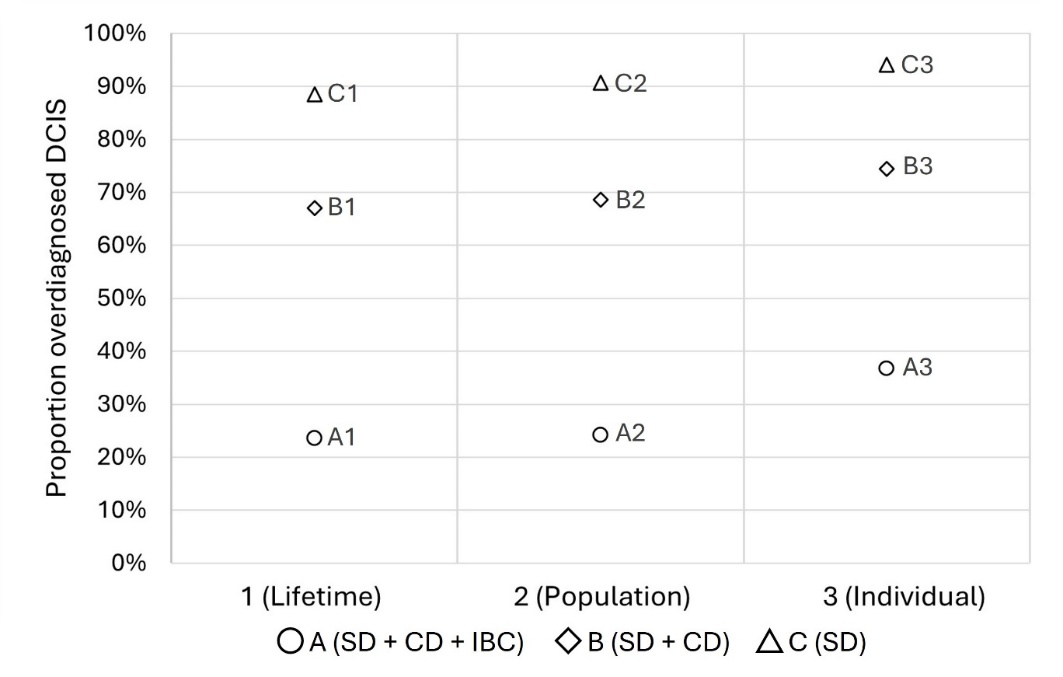


*Variation in estimated proportion DCIS overdiagnosed for variation in definition, excluding IBC from three perspectives. All proportions were calculated as number of overdiagnosed DCIS (diagnosed in screening–diagnosed without screening) divided by the total number of DCIS diagnosed in the screening scenario. Shape indicates the type of detection included as overdiagnosed. 95% Confidence intervals were reported in Appendix A.3.4. *DCIS = ductal carcinoma in situ, SD = screen-detected, CD = clinically detected, IBC = DCIS progressed to invasive breast cancer.*

###

### A.3.4. Variation in estimated proportion with 95%CI: IBC excluded

| Proportion overdiagnosed DCIS (95%CI)–Data Figure A.3.2. | | | |
| --- | --- | --- | --- |
| Definition: Overdiagnosed DCIS | Perspective | | |
|  | 1 (Lifetime) | 2 (Population) | 3 (Individual) |
| A (SD + CD + IBC) | 24% (13%–35%) | 24% (14%–33%) | 37% (27%–46%) |
| B (SD + CD) | 67% (53%–85%) | 69% (55%–86%) | 74% (62%–89%) |
| C (SD) | 89% (83%–95%) | 91% (86%–96%) | 94% (91%–98%) |

*Variation in proportion overdiagnosed DCIS, with 95% confidence intervals (addition to Figure A.3.3.). All proportions were calculated as number of overdiagnosed DCIS (diagnosed with screening–diagnosed without screening) divided by the total number of DCIS diagnosed with screening (including IBC). *DCIS = ductal carcinoma in situ, SD = screen-detected, CD = clinically detected, IBC = DCIS progressed to invasive breast cancer.*

## A.4. Univariate sensitivity analyses input

### A.4.1. Onset DCIS

| Parameter | | Base case (95%CI) | | |
| --- | --- | --- | --- | --- |
|  | Age | *Grade 1* | *Grade 2* | *Grade 3* |
| DCIS onset  (*10^-3^ /year) | 0-19 | 0.0000 (0.0000–0.0001) | 0.0000 (0.0000–0.0000) | 0.0000 (0.0000–0.0000) |
|  | 20-24 | 0.0016 (0.0014–0.0018) | 0.0003 (0.0002–0.0003) | 0.0013 (0.0012–0.0015) |
|  | 25-29 | 0.0019 (0.0017–0.0021) | 0.0043 (0.0038–0.0047) | 0.0075 (0.0067–0.0082) |
|  | 30-34 | 0.0017 (0.0015–0.0018) | 0.0127 (0.0114–0.0140) | 0.0199 (0.0179–0.0219) |
|  | 35-39 | 0.0119 (0.0107–0.0131) | 0.0221 (0.0199–0.0243) | 0.0406 (0.0365–0.0447) |
|  | 40-44 | 0.0213 (0.0191–0.0234) | 0.0516 (0.0465–0.0568) | 0.0526 (0.0474–0.0579) |
|  | 45-48 | 0.0398 (0.0358–0.0438) | 0.0600 (0.0540–0.0661) | 0.0665 (0.0598–0.0731) |
|  | 49-54 | 0.2095 (0.1885–0.2304) | 0.3105 (0.2795–0.3416) | 0.3250 (0.2925–0.3575) |
|  | 55-59 | 0.0859 (0.0773–0.0945) | 0.1805 (0.1624–0.1985) | 0.2558 (0.2302–0.2813) |
|  | 60-64 | 0.0948 (0.0853–0.1043) | 0.2396 (0.2156–0.2635) | 0.3002 (0.2702–0.3302) |
|  | 65-69 | 0.1016 (0.0915–0.1118) | 0.2674 (0.2407–0.2942) | 0.2857 (0.2571–0.3143) |
|  | 70-75 | 0.1540 (0.1386–0.1694) | 0.3556 (0.3201–0.3912) | 0.3703 (0.3332–0.4073) |
|  | 76-79 | 0.0307 (0.0277–0.0338) | 0.0630 (0.0567–0.0693) | 0.0491 (0.0442–0.0540) |
|  | 80-95 | 0.0361 (0.0324–0.0397) | 0.0585 (0.0527–0.0644) | 0.0452 (0.0407–0.0497) |
|  | 95+ | 0.0000 (0.0000–0.0013) | 0.0000 (0.0000–0.0013) | 0.0000 (0.0000–0.0026) |

*Input parameters univariate sensitivity analyses of DCIS onset with lower and upper 95% confidence intervals (95%CI).*

###

### A.4.2. DCIS progression to IBC

| Parameter | | Base case (95% CI) | | |
| --- | --- | --- | --- | --- |
|  | Age | *Grade 1* | *Grade 2* | *Grade 3* |
| Progression to IBC  (/year) | 0-19 | 0.000 (0.000–0.000) | 0.000 (0.000–0.000) | 0.000 (0.000–0.000) |
|  | 20-55 | 0.087 (0.066–0.112) | 0.137 (0.104–0.176) | 0.159 (0.121–0.205) |
|  | 55+ | 0.073 (0.056–0.093) | 0.115 (0.088–0.146) | 0.134 (0.102–0.170) |

*Input parameters univariate sensitivity analyses of DCIS progression to invasive breast cancer (IBC) with lower and upper 95% confidence intervals (95%CI).*
